# The effect of periodontal status in the associations between socioeconomic status and cognitive performance: a mediation analysis in older adults

**DOI:** 10.3389/fnagi.2025.1524268

**Published:** 2025-06-19

**Authors:** Heming Zhang, Hongxia Xiang, Lin Zhang, Zhiyang Chen, Wei Liu

**Affiliations:** ^1^Department of Anesthesiology, The Second Affiliated Hospital of Air Force Medical University, Xi’an, China; ^2^Department of Cardiology, National Center of Gerontology, Beijing Hospital, Institute of Geriatric Medicine, Chinese Academy of Medical Sciences, Beijing, China; ^3^Department of Anesthesiology, The 963 Hospital of the PLA Joint Logistics Support Force, Jiamusi, China; ^4^Department of Rheumatology and Immunology, The Second Affiliated Hospital of Air Force Medical University, Xi’an, China

**Keywords:** socioeconomic status, periodontal status, cognitive performance, NHANES, elderly adults

## Abstract

**Introduction:**

The aim of this study is to analyse the association of socioeconomic status (SES) with cognitive performance, and the mediation effect of periodontal status in this relationship in the National Health and Nutrition Examination Survey (NHANES) database from 2011–2014.

**Methods:**

The SES was evaluated based on poverty-income ratio (PIR), occupation, educational level, and health insurance using latent class analysis. Multivariable logistic regressions were used to determine the association of cognitive performance, examined by Consortium to Establish a Registry for Alzheimer’s Disease (CERAD) test, animal fluency test (AFT), and digit symbol substitution test (DSST), with SES, attachment loss (AL) and probing depth (PD). Multivariable linear regressions were used to explore the association of mean AL and mean PD with SES. A mediation analysis was conducted to examine the impact of mean AL and mean PD on the relationship between SES and cognitive performance.

**Results:**

The study included 1,812 participants aged 60 years or older. In the fully adjusted model, SES showed a positive correlation with all three cognitive tests. Meanwhile, mean AL [odds ratio (OR) = 1.61; 95% confidence interval (CI): 1.33 to 1.95] and mean PD (OR = 2.14; 95% CI: 1.54 to 2.96) were inversely related to the DSST scores, accounting for 12.17 and 6.91% of the relationship between SES and DSST, respectively. The mediation effect of periodontal status in this association was significant only in non-HSB participants or in younger participants.

**Conclusion:**

SES was negatively associated with periodontal status in older adults in the United States. Furthermore, the link between SES and cognitive performance can be partially explained by periodontal status.

## 1 Introduction

The global population is rapidly ageing, attributable to increases in life expectancy (Pais et al., 2020). In 2019, approximately 9% of the population in the world was over 65 years old, and this percentage will increase to 16% by 2050 (Huang and Ren, 2022). Age-related cognitive decline is emerging as a significant health concern among the elderly. In the United States, around 36% of the population experiences cognitive impairment, a rate anticipated to double by 2050 (Hebert et al., 2013). Cognitive impairment imposes substantial emotional and financial strains on individuals and healthcare systems.

Socioeconomic status (SES), a crucial determinant of health outcomes, has been shown to positively correlate with cognitive performance (Liu et al., 2022). However, the underlying mechanisms of this relationship remain unclear. One hypothesis is that lower SES is associated with higher levels of systemic inflammation, which may further contribute to cognitive decline (Muscatell et al., 2020). However, the evidence linking SES and inflammation is inconsistent, potentially due to variations in the measures of SES and sources of inflammation (Muscatell et al., 2020).

Periodontitis, a significant oral health condition that manifests throughout the human lifespan, is marked by a polymicrobial dysbiotic infection of the periodontium (Ebersole et al., 2018; Hajishengallis, 2015). Research has identified certain pathogens involved in periodontitis as contributing factors to the pathogenesis of Alzheimer’s disease (AD) (Honjo et al., 2009). Evidence suggested that participants aged 20–59 years with periodontitis had significantly higher scores on the symbol digit substitution test and serial digit learning test (Sung et al., 2019). Recent systematic reviews and meta-analyses further consolidated evidence linking periodontitis to cognitive disorders, reporting pooled odds ratios of approximately 1.23 for general cognitive decline (Dibello et al., 2024). Furthermore, SES has been highlighted as a potential etiological factor for periodontitis (Lenk et al., 2022; Han et al., 2019). Nevertheless, these studies often used a single variable to represent SES, providing an incomplete depiction of its impact. The role of periodontal status in the relationship between SES and cognitive performance remains unclear.

Therefore, we implemented a cross-sectional study to assess the potential correlation of SES with periodontal status and cognitive performance among older adults from the National Health and Nutrition Examination Survey (NHANES) 2011–2014 cohort. Additionally, we conducted further analysis to evaluate whether periodontal status could mediate the relationship between SES and cognitive performance.

## 2 Method

### 2.1 Data sources

Data were extracted from the NHANES database, a nationally representative cross-sectional survey conducted by the United States Centers for Disease Control (CDC). The Research Ethics Review Board of the National Center for Health Statistics (NCHS) approved the survey protocol. All individuals provided written informed consent before participating in the study (accessible at https://www.cdc.gov/nchs/nhanes/irba98.html).

### 2.2 Study population

**Figure 1:**
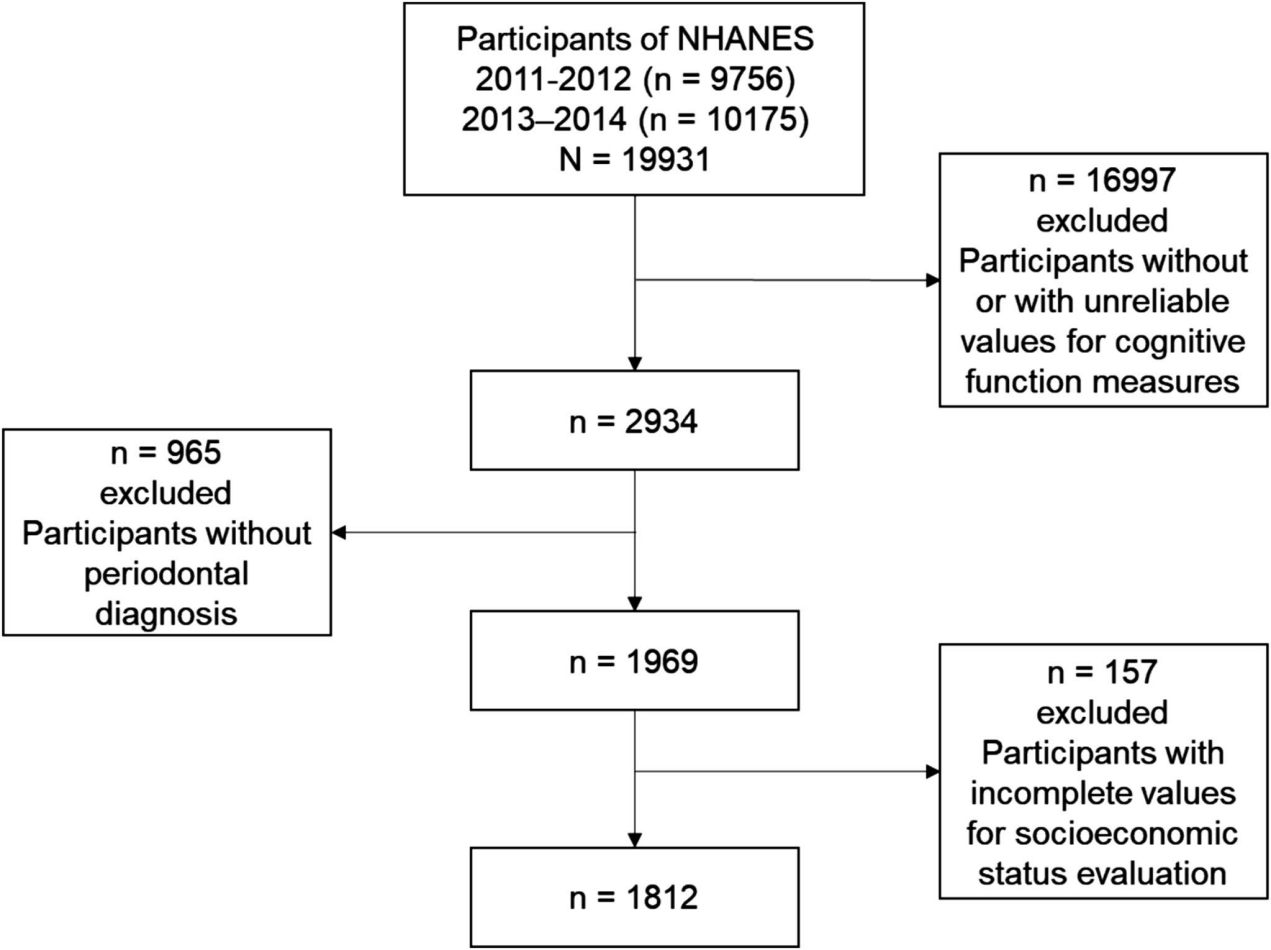
Flow chart of the screening process for the selection of eligible participants.

Initially, this study encompassed 19,931 participants across two cycles from 2011 to 2014. Participants were included if they met the following criteria: (1) they were aged 60 years or older; (2) they had reliable values for cognitive function measures; (3) they had periodontal diagnosis data; and (4) they had complete data for socioeconomic status evaluation. After applying these criteria, a total of 1,812 NHANES participants were included in the final analysis (Figure 1).

### 2.3 Cognitive test battery

Cognitive performance was evaluated using the Consortium to Establish a Registry for Alzheimer’s Disease (CERAD) test, the animal fluency test (AFT), and the digit symbol substitution test (DSST). The CERAD test comprises three consecutive learning trials and one delayed recall to assess episodic memory (Bailey et al., 2020). After learning, participants were required to recall as many words as possible, with the cumulative score constituting the CERAD score. The AFT assessed verbal fluency and semantic-based memory function (Bailey et al., 2020). Participants who passed the sample practice pretest were asked to name as many animals as possible in 1 min. The DSST was used to evaluate the processing speed, sustained attention, and working memory of participants. Participants had 2 min to copy the corresponding symbols in the 133 boxes that adjoin the numbers (Brody et al., 2019). The number of correct matches determines the final DSST score. Based on prior studies, participants were categorized into two groups: those with low cognitive performance and those with normal cognitive performance, with cutoff points established based on participants’ ages (Supplementary Table 1).

### 2.4 Periodontal assessment

Periodontal examination was conducted as part of an oral health program according to the NHANES Oral Health Examiners Manual. Attachment loss (AL) and probing depth (PD) were recorded at six sites per tooth and participants with fewer than two teeth were excluded.

### 2.5 Socioeconomic status assessment

The poverty income ratio (PIR), educational level, occupation, and health insurance status were combined to formulate an overall socioeconomic status (SES) parameter (Krueger et al., 2025). These variables were divided into three levels based on practical interpretation (Table 1). The SES parameter was derived using latent class analysis, with the maximum absolute deviation established at 1 × 10^−10^. The Akaike information criterion (AIC), Bayesian information criterion (BIC), and *G*^2^ served as benchmarks for model selection.

**Table 1:** The classifications of variables related to socioeconomic status.

Covariates	Classifications
PIR	<1.3; 1.3 to < 3.5; ≥3.5
Occupation	Upper (socioeconomic index ≥50); lower (socioeconomic index <50, including retirees and students); unemployment
Educational level	Below high school (less than 9th grade or 9–11th grade including 12th grade with no diploma); high school (high school graduate, GED, or equivalent); college or above (college, AA degree or above)
Health insurance	Private health insurance (private health insurance, Medi-gap, or single-service plan); public health insurance (Medicare, Medicaid, State Children’s Healthcare Plan, military healthcare, Indian Health Service, State Sponsored Health Plan, or other government program); no health insurance

PIR, poverty income ratio.

### 2.6 Covariates

The potential confounding factors examined in this study included sex (male and female); age; body mass index (BMI) categories (<25; 25 to <30; ≥30); race (Non-Hispanic White, Non-Hispanic Black, and Other); marital status (never married, married, divorced, widowed, and other); smoking (yes and no); alcohol consumption (yes and no); work and recreational activities (vigorous or moderate, and other); and health conditions including clinical depression, diabetes, hypertension, and stroke.

More specifically, smoking status was determined based on participants’ self-reports. Alcohol consumption was defined as having at least 12 alcoholic beverages per year. Depression was assessed with the Patient Health Questionnaire-9 (PHQ-9), scoring responses from “not at all” to “nearly every day” on a scale of 0 to 3. Participants with PHQ-9 ≥ 10 were considered to have clinical depression. Diabetes, hypertension, and stroke were identified if participants had ever been diagnosed with these conditions by a healthcare provider.

### 2.7 Statistical analysis

All statistical analyses were conducted using R version 4.2.1. In accordance with the analytical guidelines provided by the NHANES, we constructed the new sample weights (the original 2-year sample weight divided by 2). Missing data were assessed using the package “VIM” and complemented by multiple imputation. Counting data were summarized by the count and percentage [*n* (%)] and assessed using either the chi-square (*χ*^2^) test or Fisher’s exact test as appropriate.

Multiple logistic regression analysis was employed to investigate the association of low cognitive performance with SES, mean AL, and mean PD. The findings were presented as odds ratios (OR) with corresponding 95% confidence interval (CI). Multiple linear regression was utilized to examine the association of mean AL and mean PD with SES, with results reported as β and 95% CI. In our analysis, Model 1 was unadjusted for confounders. Model 2 accounted for sex, age, body mass index (BMI), race, and marital status. Model 3 was further adjusted for smoking, alcohol consumption, work activity, creative activities, depression, diabetes, hypertension, and stroke based on Model 2. Mediation analysis was conducted based on R package “boot” (Supplementary Figure 1). The mediation effect of mean AL and mean PD in different subgroups divided by sex, age, BMI, race, smoking, alcohol consumption, and physical activity in model 3 was further analyzed. A two-sided *p*-value of less than 0.05 was considered statistically significant.

## 3 Results

From 2,934 participants who had complete and reliable cognitive performance test data, 1,812 with complete periodontal data were included in our analyses (Figure 1). The proportion of missing data for selected variables did not exceed 10% of the total sample. Based on the results of AIC, BIC, and *G*^2^, participants were categorized into three SES groups (Supplementary Figure 2 and Table 2; Supplementary Table 2).

**Table 2:** Practical definitions of high, medium, and low socioeconomic status.

Insurance Type	Education Level	upper socioeconomic index			lower socioeconomic index			unemployment		
		PIR≥3.5	PIR≥1.3 to < 3.5	PIR<1.3	PIR≥3.5	PIR≥1.3 to < 3.5	PIR<1.3	PIR≥3.5	PIR≥1.3 to < 3.5	PIR<1.3
Private health insurance	College or above	3	3	3	3	2	2	3	2	2
	High school	3	2	2	2	2	2	2	2	2
	Below high school	2	2	NA	2	2	2	2	2	1
Public health insurance	College or above	3	3	3	3	2	1	3	2	1
	High school	3	NA	2	2	2	1	2	2	1
	Below high school	NA	NA	NA	2	2	1	2	1	1
No health insurance	College or above	3	3	2	2	2	1	2	1	1
	High school	NA	2	NA	2	2	1	2	1	1
	Below high school	NA	NA	1	2	1	1	NA	1	1

PIR, poverty income ratio. 3 = High SES; 2 = Medium SES; 1 = Low SES; NA = no participants in this group.

Table 3 displays the baseline characteristics of the study cohort (*n* = 1,812). Statistically significant differences were observed among the participants in different groups according to sex (*p* = 0.01), age (*p* < 0.01), race (*p* < 0.01), marital status (*p* < 0.01), smoking status (*p* = 0.03), alcohol consumption (*p* < 0.01), physical activity (*p* < 0.01), clinical depression (*p* < 0.01), hypertension (*p* < 0.01), diabetes (*p* < 0.01), and stroke (*p* < 0.01), but not according to BMI.

**Table 3:** Characteristics of the study population, National Health and Nutrition Examination Survey (NHANES) 2011–2014 (*N* = 1812).

Characteristics	Participants, No. (%)				*p*-value
	Total sample	Low SES	Medium SES	High SES	
	(*N* = 1812)	(*n* = 370)	(*n* = 909)	(*n* = 533)	
Sex, *n* (%)					0.01
Male	898 (49.56)	171 (46.22)	450 (49.5)	277 (51.97)	
Female	914 (50.44)	199 (53.78)	459 (50.5)	256 (48.03)	
Age (years), *n* (%)					<0.01
60–69	1,086 (59.93)	269 (72.7)	494 (54.35)	323 (60.6)	
70–79	480 (26.49)	69 (18.65)	270 (29.7)	141 (26.45)	
≥80	246 (13.58)	32 (8.65)	145 (15.95)	69 (12.95)	
BMI (kg/m^2^), *n* (%)					0.15
<25	483 (26.66)	103 (27.84)	234 (25.74)	146 (27.39)	
25 to <30	655 (36.15)	116 (31.35)	323 (35.53)	216 (40.53)	
≥30	674 (37.2)	151 (40.81)	352 (38.72)	171 (32.08)	
Race, *n* (%)					<0.01
NHB	413 (22.79)	109 (29.46)	220 (24.2)	84 (15.76)	
NHW	861 (47.52)	82 (22.16)	457 (50.28)	322 (60.41)	
Other	538 (29.69)	179 (48.38)	232 (25.52)	127 (23.83)	
Marital status, *n* (%)					<0.01
Never married	108 (5.96)	41 (11.08)	43 (4.73)	24 (4.5)	
Married	1,043 (57.56)	139 (37.57)	522 (57.43)	382 (71.67)	
Divorced	258 (14.24)	69 (18.65)	131 (14.41)	58 (10.88)	
Widowed	296 (16.34)	82 (22.16)	166 (18.26)	48 (9.01)	
Other	107 (5.91)	39 (10.54)	47 (5.17)	21 (3.94)	
Smoking, *n* (%)					0.03
No	869 (47.96)	181 (48.92)	461 (50.72)	227 (42.59)	
Yes	943 (52.04)	189 (51.08)	448 (49.28)	306 (57.41)	
Alcohol consumption, *n* (%)					<0.01
No	553 (30.52)	143 (38.65)	300 (33)	110 (20.64)	
Yes	1,259 (69.48)	227 (61.35)	609 (67)	423 (79.36)	
Physical activity, *n* (%)					<0.01
Vigorous or moderate	1,136 (62.69)	179 (48.38)	563 (61.94)	394 (73.92)	
Other	676 (37.31)	191 (51.62)	346 (38.06)	139 (26.08)	
Clinical depression, *n* (%)	1,673 (92.33)	314 (84.86)	843 (92.74)	516 (96.81)	<0.01
Hypertension, *n* (%)	1,078 (59.49)	240 (64.86)	557 (61.28)	281 (52.72)	<0.01
Diabetes, *n* (%)	391 (21.58)	110 (29.73)	208 (22.88)	73 (13.7)	<0.01
Stroke, *n* (%)	91 (5.02)	29 (7.84)	47 (5.17)	15 (2.81)	<0.01
CERAD, *n* (%)					<0.01
Normal cognitive performance	1,409 (77.76)	230 (62.16)	707 (77.78)	472 (88.56)	
Low cognitive performance	403 (22.24)	140 (37.84)	202 (22.22)	61 (11.44)	
AFT, *n* (%)					<0.01
Normal cognitive performance	1,364 (75.28)	206 (55.68)	696 (76.57)	462 (86.68)	
Low cognitive performance	448 (24.72)	164 (44.32)	213 (23.43)	71 (13.32)	
DSST, *n* (%)					<0.01
Normal cognitive performance	1,370 (75.61)	168 (45.41)	696 (76.57)	506 (94.93)	
Low cognitive performance	442 (24.39)	202 (54.59)	213 (23.43)	27 (5.07)	

NHW, Non-Hispanic White; NHB, Non-Hispanic Black; BMI, body mass index; CERAD, Consortium to Establish a Registry for Alzheimer’s Disease; AFT, animal fluency test; DSST, digit symbol substitution test.

The associations between SES and cognitive performance were presented in Table 4. Compared with participants in the low SES group, those in the high SES group exhibited a lower likelihood of experiencing low cognitive performance as measured by CERAD (Model 1, OR = 0.17, 95% CI: 0.1 to 0.31; Model 2, OR = 0.18, 95% CI: 0.1 to 0.31; Model 3, OR = 0.22, 95% CI: 0.12 to 0.4), AFT (Model 1, OR = 0.13, 95% CI: 0.08 to 0.22; Model 2, OR = 0.19, 95% CI: 0.12 to 0.29; Model 3, OR = 0.25, 95% CI: 0.15 to 0.41), and DSST (Model 1, OR = 0.03, 95% CI: 0.02 to 0.05; Model 2, OR = 0.06, 95% CI: 0.03 to 0.1; Model 3, OR = 0.08, 95% CI: 0.04 to 0.14) in all three models.

**Table 4:** Associations of SES with low cognitive performance.

Test	SES Category	Model 1		Model 2		Model 3	
		OR (95% CI)	*p*-value	OR (95% CI)	*p*-value	OR (95% CI)	*p*-value
CERAD	Low SES	referent	referent	referent	referent	referent	referent
	Medium SES	0.43 (0.29 to 0.63)	< 0.01	0.44 (0.29 to 0.68)	< 0.01	0.47 (0.3 to 0.75)	< 0.01
	High SES	0.17 (0.1 to 0.31)	< 0.01	0.18 (0.1 to 0.31)	< 0.01	0.22 (0.12 to 0.4)	< 0.01
AFT	Low SES	referent	referent	referent	referent	referent	referent
	Medium SES	0.33 (0.2 to 0.54)	< 0.01	0.45 (0.27 to 0.73)	< 0.01	0.52 (0.3 to 0.89)	0.02
	High SES	0.13 (0.08 to 0.22)	< 0.01	0.19 (0.12 to 0.29)	< 0.01	0.25 (0.15 to 0.41)	< 0.01
DSST	Low SES	referent	referent	referent	referent	referent	referent
	Medium SES	0.18 (0.13 to 0.27)	< 0.01	0.24 (0.16 to 0.36)	< 0.01	0.27 (0.18 to 0.42)	< 0.01
	High SES	0.03 (0.02 to 0.05)	< 0.01	0.06 (0.03 to 0.1)	< 0.01	0.08 (0.04 to 0.14)	< 0.01

OR, odds ratio; CI, confidence interval; CERAD, Consortium to Establish a Registry for Alzheimer’s Disease; AFT, animal fluency test; DSST, digit symbol substitution test; SES, socioeconomic status. Model 1: not adjusted for covariates. Model 2: adjusted for age, sex, race, marital status, and BMI. Model 3: Model 2 + smoking, alcohol consumption, physical activities, clinical depression, diabetes, hypertension, and stroke.

The relationship between periodontal status and cognitive performance was shown in Table 5. In Model 1, mean AL and mean PD were positively associated with all three cognitive tests. However, when all covariates were adjusted, mean AL was only positively associated with AFT [OR with 95% CI of 1.21 (1.02 to 1.43)] and DSST [OR with 95% CI of 1.61 (1.33 to 1.95)]. Mean PD was significantly associated with CERAD [OR with 95% CI of 1.36 (1.05 to 1.77)] and DSST [OR with 95% CI of 2.14 (1.54 to 2.96)] but not with AFT.

**Table 5:** Associations of mean AL, mean PD, and periodontitis with low cognitive performance.

Test	Periodontal status	Model 1		Model 2		Model 3	
		OR (95% CI)	P-Value	OR (95% CI)	P-Value	OR (95% CI)	P-Value
CERAD	Mean AL	1.3 (1.12 to 1.5)	< 0.01	1.18 (0.99 to 1.4)	0.06	1.17 (0.97 to 1.41)	0.09
	Mean PD	1.67 (1.39 to 2.01)	< 0.01	1.37 (1.1 to 1.71)	0.01	1.36 (1.05 to 1.77)	0.02
AFT	Mean AL	1.36 (1.19 to 1.56)	< 0.01	1.22 (1.05 to 1.42)	0.01	1.21 (1.02 to 1.43)	0.03
	Mean PD	1.49 (1.12 to 2)	0.01	1.1 (0.79 to 1.53)	0.54	1.07 (0.76 to 1.51)	0.66
DSST	Mean AL	1.84 (1.57 to 2.16)	< 0.01	1.63 (1.38 to 1.93)	< 0.01	1.61 (1.33 to 1.95)	< 0.01
	Mean PD	3.06 (2.27 to 4.12)	< 0.01	2.15 (1.56 to 2.97)	< 0.01	2.14 (1.54 to 2.96)	< 0.01

OR, odds ratio; CI, confidence interval; AL, attachment loss; PD, probing depth. Model 1: not adjusted for covariates. Model 2: adjusted for age, sex, race, marital status, and BMI. Model 3: Model 2 + smoking, alcohol consumption, physical activities, clinical depression, diabetes, hypertension, and stroke.

Table 6 showed the associations of SES with mean AL and mean PD. Compared with participants in the low SES group, those in the high SES group showed an inverse associated with mean AL (Model 1, OR = −1.06, 95% CI: −1.31 to −0.81; Model 2, OR = −0.85, 95% CI: −1.14 to −0.56; Model 3, OR = −0.81, 95% CI: −1.15 to −0.46) and mean PD (Model 1, OR = −0.39, 95% CI: −0.5 to −0.27; Model 2, OR = −0.29, 95% CI: −0.42 to −0.16; Model 3, OR = −0.3, 95% CI: −0.45 to −0.15) in all three models.

**Table 6:** Associations of SES with mean AL and mean PD.

Periodontal status	SES Category	Model 1		Model 2		Model 3	
		β (95% CI)	*p*-value	β (95% CI)	*p*-value	β (95% CI)	*p*-value
Mean AL	Low SES	referent	referent	referent	referent	referent	referent
	Medium SES	-0.61 (-0.81 to -0.4)	< 0.01	-0.44 (-0.67 to -0.21)	< 0.01	-0.44 (-0.71 to -0.18)	0.01
	High SES	-1.06 (-1.31 to -0.81)	< 0.01	-0.85 (-1.14 to -0.56)	< 0.01	-0.81 (-1.15 to -0.46)	< 0.01
Mean PD	Low SES	referent	referent	referent	referent	referent	referent
	Medium SES	-0.27 (-0.37 to -0.17)	< 0.01	-0.18 (-0.3 to -0.07)	< 0.01	-0.19 (-0.32 to -0.06)	0.01
	High SES	-0.39 (-0.5 to -0.27)	< 0.01	-0.29 (-0.42 to -0.16)	< 0.01	-0.3 (-0.45 to -0.15)	< 0.01

CI, confidence interval; AL, attachment loss; PD, probing depth; SES, socioeconomic status. Model 1: not adjusted for covariates. Model 2: adjusted for age, sex, race, marital status, and BMI. Model 3: Model 2 + smoking, alcohol consumption, physical activities, clinical depression, diabetes, hypertension, and stroke.

The mediating effect of mean AL on the correlation between SES and cognitive performance is presented in Table 7. In model 1, mean AL could mediate the association of SES with cognitive performance in all three cognitive tests. However, in the fully adjusted model, mean AL could only mediate 11.05% of the association between SES and DSST [β with 95% CI of −0.25 (−0.41 to −0.13)]. Similarly, the mediation effect of mean PD was only significant in the association of SES with DSST in model 3 [β with 95% CI of −0.16 (−0.3 to −0.06)] (Table 8).

**Table 7:** The mediating proportion of mean AL on the association between SES and low cognitive performance.

Test	Model	Direct Effect		Indirect Effect		Total Effect		Proportion Mediated (%)
		β (95%CI)	*p*-value	β (95%CI)	*p*-value	β (95%CI)	*p*-value	
CERAD	Model 1	-1.62 (-2.13 to -1.13)	< 0.01	-0.13 (-0.27 to 0)	0.028	-1.75 (-2.24 to -1.28)	< 0.01	7.60%
	Model 2	-1.67 (-2.22 to -1.16)	< 0.01	-0.04 (-0.18 to 0.08)	0.245	-1.71 (-2.25 to -1.25)	< 0.01	-
	Model 3	-1.46 (-2.02 to -0.93)	< 0.01	-0.05 (-0.18 to 0.07)	0.183	-1.51 (-2.08 to -1.07)	< 0.01	-
AFT	Model 1	-1.85 (-2.33 to -1.37)	< 0.01	-0.17 (-0.34 to -0.03)	0.011	-2.02 (-2.52 to -1.56)	< 0.01	8.53%
	Model 2	-1.61 (-2.15 to -1.15)	< 0.01	-0.08 (-0.22 to 0.07)	0.134	-1.69 (-2.21 to -1.25)	< 0.01	-
	Model 3	-1.31 (-1.86 to -0.81)	< 0.01	-0.09 (-0.25 to 0.06)	0.135	-1.4 (-2.01 to -0.95)	< 0.01	-
DSST	Model 1	-2.99 (-3.69 to -2.46)	< 0.01	-0.46 (-0.64 to -0.31)	< 0.01	-3.39 (-4.07 to -2.85)	< 0.01	13.58%
	Model 2	-2.58 (-3.34 to -2.05)	< 0.01	-0.31 (-0.47 to -0.17)	< 0.01	-2.86 (-3.56 to -2.34)	< 0.01	10.72%
	Model 3	-2.27 (-3.06 to -1.72)	< 0.01	-0.31 (-0.48 to -0.17)	< 0.01	-2.54 (-3.34 to -2.01)	< 0.01	12.17%

CI, confidence interval; CERAD, Consortium to Establish a Registry for Alzheimer’s Disease; AFT, animal fluency test; DSST, digit symbol substitution test. Model 1: not adjusted for covariates. Model 2: adjusted for age, sex, race, marital status, and BMI. Model 3: Model 2 + smoking, alcohol consumption, physical activities, clinical depression, diabetes, hypertension, and stroke.

**Table 8:** The mediating proportion of mean PD on the association between SES and low cognitive performance.

Test	Model	Direct Effect		Indirect Effect		Total Effect		Proportion Mediated (%)
		β (95%CI)	*p*-value	β (95%CI)	*p*-value	β (95%CI)	*p*-value	
CERAD	Model 1	-1.64 (-2.16 to -1.16)	< 0.01	-0.11 (-0.24 to -0.01)	0.016	-1.75 (-2.29 to -1.28)	< 0.01	6.50%
	Model 2	-1.67 (-2.22 to -1.19)	< 0.01	-0.04 (-0.14 to 0.04)	0.152	-1.71 (-2.24 to -1.24)	< 0.01	-
	Model 3	-1.46 (-1.97 to -0.96)	< 0.01	-0.05 (-0.15 to 0.04)	0.105	-1.51 (-2.08 to -1.06)	< 0.01	-
AFT	Model 1	-1.97 (-2.45 to -1.54)	< 0.01	-0.05 (-0.16 to 0.07)	0.176	-2.02 (-2.47 to -1.58)	< 0.01	-
	Model 2	-1.71 (-2.26 to -1.26)	< 0.01	0.02 (-0.07 to 0.13)	0.705	-1.69 (-2.17 to -1.25)	< 0.01	-
	Model 3	-1.42 (-1.99 to -0.94)	< 0.01	0.02 (-0.08 to 0.15)	0.66	-1.4 (-1.92 to -0.97)	< 0.01	-
DSST	Model 1	-3.14 (-3.91 to -2.64)	< 0.01	-0.3 (-0.44 to -0.19)	< 0.01	-3.39 (-4.03 to -2.85)	< 0.01	8.91%
	Model 2	-2.73 (-3.48 to -2.19)	< 0.01	-0.16 (-0.28 to -0.07)	< 0.01	-2.86 (-3.63 to -2.31)	< 0.01	5.68%
	Model 3	-2.4 (-3.17 to -1.83)	< 0.01	-0.18 (-0.3 to -0.07)	0.001	-2.54 (-3.33 to -2)	< 0.01	6.91%

CI, confidence interval; CERAD, Consortium to Establish a Registry for Alzheimer’s Disease; AFT, animal fluency test; DSST, digit symbol substitution test. Model 1: not adjusted for covariates. Model 2: adjusted for age, sex, race, marital status, and BMI. Model 3: Model 2 + smoking, alcohol consumption, physical activities, clinical depression, diabetes, hypertension, and stroke.

The mediation effect of mean AL and mean PD in different subgroups were analyzed. After adjusting for all covariates, mean AL could only mediate the relationship between SES and CERAD in participants with BMI less than 25 and the relationship between SES and AFT in male participants (Supplementary Tables 3, 4). In terms of DSST, mean AL could mediate this association in all participants except for those older than 80 or NHB participants (Supplementary Table 5). Moreover, mean PD could only mediate the relationship between SES and CERAD in participants with BMI less than 25 or those in the vigorous/moderate group (Supplementary Table 6). No significant mediation effect of mean PD was observed in the relationship between SES and AFT (Supplementary Table 7). Regarding DSST, mean PD could mediate this correlation in all participants except for those aged older than 70, those with BMI less than 30, NHB participants, or those with no alcohol consumption (Supplementary Table 8).

## 4 Discussion

In this study, the relationship between SES and periodontal status and the mediation effect of periodontal status in the association between SES and cognitive performance in participants aged 60 years or older in the United States was investigated. The results showed that the association of SES with cognitive performance, particularly in terms of DSST performance, could be partly mediated by periodontal status. Moreover, there were differences in the mediating effects of periodontal status in this association among different subgroups, particularly in terms of age and race.

Socioeconomic inequity in cognitive decline has been widely discussed. A 2025 JAMA Network Open cohort of 7,303 adults demonstrated that higher adulthood SES was associated with slower cognitive decline and more favorable brain MRI markers, including reduced white matter hyperintensities and greater total brain volume (Krueger et al., 2025). Besides, a previous study based on National Longitudinal Study of Adolescent to Adult Health indicated that participants with high SES, particularly those with occupations that use analytical skills and involve forms of social interaction, were more likely to have better cognitive performance (Stebbins et al., 2022). Furthermore, a meta-analysis of 39 prospective studies reported that low-SES participants had a 31% higher risk of cognitive impairment and dementia compared with high-SES counterparts (Wang et al., 2023). However, several potential reasons leading to the important gaps remain. First, SES was a complex concept which cannot be represented by a single indicator (Petrovic et al., 2018). In addition, the characteristics of study populations, design, and data collection methods varied widely. In this study, we constructed a comprehensive SES variable including PIR, educational level, occupation, and health insurance, and confirmed the socioeconomic disparity in cognitive performance. Notably, periodontal measures demonstrated significant associations with all three cognitive outcomes in unadjusted analyses. However, these associations were attenuated for CERAD and AFT following adjustment for potential confounders, while the relationship with DSST remained robust. This attenuation reflects the multifactorial nature of both periodontal disease and cognitive decline. In particular, the mediation effect of periodontal measures was robust for DSST outcomes, suggesting that periodontal health is particularly relevant to cognitive domains related to processing speed, sustained attention, and working memory. Conversely, the mediation effects observed for the CERAD and AFT tests were weaker or only evident in certain subgroups. The differential findings across cognitive tests may be due to the inherent differences in what each cognitive test measures and their sensitivity to inflammation-driven cognitive decline (Sung et al., 2019; Li et al., 2021).

There have been several possible mechanisms to explain the mediation effect of periodontal status in the relationship between SES and cognitive performance in older adults. First, SES could directly affect oral health behaviors and mental health status, and poor mental health status could further exacerbate poor oral health behaviors (Meyer et al., 2014; Holde et al., 2018). Moreover, *P. gingivalis* periodontal infection could cause cognitive decline by releasing proinflammatory cytokines, including tumor necrosis factor-alpha (TNF-α), interleukin (IL)-6, and IL-1β, in the brain (Ding et al., 2018). Chronic exposure to *P. gingivalis* lipopolysaccharide could induce peripheral Aβ accumulation in inflammatory monocytes/macrophages (Nie et al., 2019). Huang et al. (2025) systematically demonstrated that *P. gingivalis-derived* outer membrane vesicles (OMVs) traverse the blood-brain barrier and induce microglial activation through TLR4-dependent signaling pathways, thus establishing a direct connection between periodontal infection and neuroinflammatory processes in the central nervous system. Shawkatova et al. (2025) identified pathogenic gingipains as catalytic mediators of tau protein and amyloid precursor protein proteolysis, thereby delineating specific enzymatic pathways through which periodontal microbiota may contribute to AD progression. Besides, Li et al. (2021) found that white blood cell count could mediate the relationship between periodontal status and cognitive function. In addition, evidence suggested that mitochondrial dysfunction was a mediating factor in the link between periodontal status and cognitive impairment (Li et al., 2022). Meanwhile, the result of this study showed that periodontal status could significantly mediate the association of SES with processing speed, sustained attention, and working memory as measured by DSST. This mediation effect of periodontal status was different among subgroups, particularly those divided by age and race, in terms of both mean AL and mean PD. Mechanistically, age-related immunosenescence and dysregulated inflammatory responses could attenuate the association between chronic periodontal inflammation and systemic/neural changes (Fulop et al., 2017). In addition, a survival bias may be at work—individuals with greater inherent resilience to both oral and systemic health challenges are more likely to live beyond 80, thereby diminishing observable SES-related differences in both periodontal health and cognitive performance (Tsakos et al., 2011). The underlying mechanisms for this race-based difference were intricate. One possible explanation could be the genetic susceptibility of periodontal disease (Goncalves-Anjo et al., 2022). African Americans may have a higher genetic susceptibility to periodontal disease, which leads to more severe symptoms of periodontal disease even under similar socioeconomic conditions (Schuch et al., 2017). This genetic susceptibility may mask the influence of socioeconomic status.

This study presents several advantages. Firstly, the data used in this study had a large sample size and excellent quality control, which increased the statistical power to provide more reliable results. Moreover, we constructed a comprehensive SES variable including four aspects to more completely represent overall SES. However, a few limitations should be noted in this study. First, it was difficult to ascertain causality due to the cross-sectional study design. In addition, the cognitive performance measures in this study could not replace a diagnosis based on a clinical examination due to the limitation of NHANES data measured cognitive performance domains.

## 5 Conclusion

This study suggested that SES was negatively associated with periodontal status in participants aged 60 years or older in the United States. The associations between SES and cognitive performance can be partly explained by periodontal status. Moreover, the mediation effect of periodontal status in this association was significant only in non-HSB participants or in younger participants. This study might help in understanding the mediation effect of periodontitis in the correlation between SES and cognitive performance, and further support the need for preventative approaches to reduce periodontitis.

## Data Availability

Publicly available datasets were analyzed in this study. This data can be found here: https://www.cdc.gov/nchs/nhanes/index.htm.
