# Supplementary material for: The effect of periodontal status in the associations between socioeconomic status and cognitive performance: a mediation analysis in older adults
Source: Front Aging Neurosci. 2025 Jun 19;17:1524268. doi: 10.3389/fnagi.2025.1524268 (PMC12222096; doi:10.3389/fnagi.2025.1524268)
Supplement: Supplementary file 1 [file Data_Sheet_1.docx]

Supplementary Material

# Supplementary Figures and Tables

## Supplementary Figures


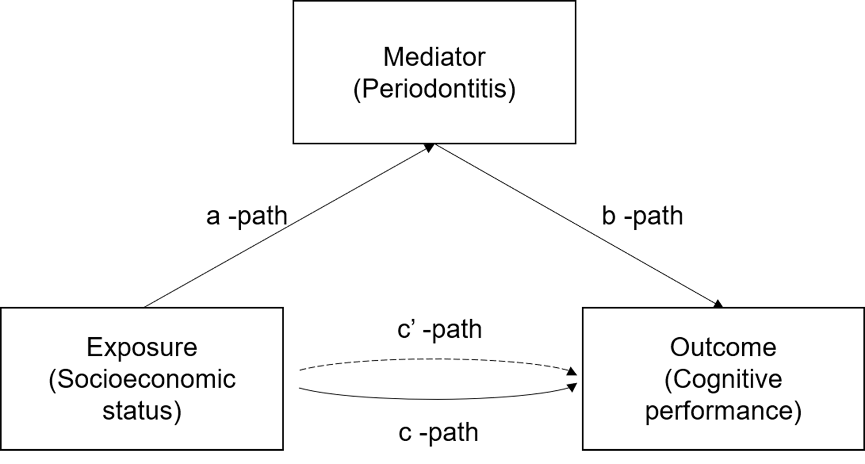


**Supplementary Figure 1** Path diagram of the mediation analysis models.


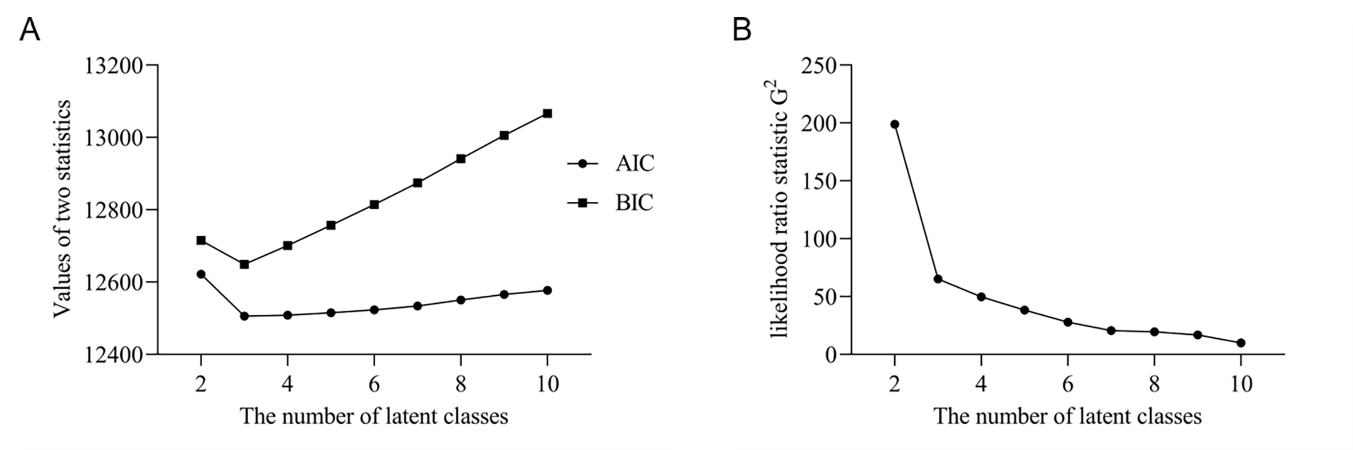


**Supplementary Figure 2** (A) AIC, BIC, and (B) G^2^ in models with different numbers of latent classes in the US NHANES.

## Supplementary Tables

**Supplementary Table 1** The cognition performance cut-off points of test score, adjusted according to age.

|  | CERAD score | AFT score | DSST score |
| --- | --- | --- | --- |
| 60–69 years | 23 | 15 | 39 |
| 70–79 years | 20 | 12 | 35 |
| ≥80 years | 17 | 11 | 30 |

Abbreviations: CERAD, Consortium to Establish a Registry for Alzheimer’s disease; AFT, animal fluency test; DSST, Digit Symbol Substitution test.

**Supplementary Table S2:** Mean posterior probabilities, prevalence of latent classes, and item-response probabilities in models with two to four latent classes in the US NHANES*

| Item† | Latent class 1 | Latent class 2 | Latent class 3 | Latent class 4 |
| --- | --- | --- | --- | --- |
| Two-latent-class solution | | | | |
| Mean PP | 0.62 | 0.38 | NA | NA |
| Prevalence | 0.58 | 0.42 | NA | NA |
| Income 1 | 0.06 | **0.51** | NA | NA |
| Income 2 | 0.37 | 0.44 | NA | NA |
| Income 3 | **0.57** | 0.05 | NA | NA |
| Occupation 1 | 0.06 | 0.27 | NA | NA |
| Occupation 2 | **0.78** | **0.73** | NA | NA |
| Occupation 3 | 0.16 | 0 | NA | NA |
| Education 1 | 0.04 | **0.44** | NA | NA |
| Education 2 | 0.19 | 0.28 | NA | NA |
| Education 3 | **0.77** | 0.28 | NA | NA |
| Insurance 1 | 0.03 | 0.17 | NA | NA |
| Insurance 2 | 0.18 | **0.58** | NA | NA |
| Insurance 3 | **0.8** | 0.26 | NA | NA |
| Three-latent-class solution | | | | |
| Mean PP | 0.2 | 0.29 | 0.5 | NA |
| Prevalence | 0.19 | 0.29 | 0.52 | NA |
| Income 1 | **0.75** | 0.04 | 0.2 | NA |
| Income 2 | 0.25 | 0.18 | **0.57** | NA |
| Income 3 | 0 | **0.78** | 0.23 | NA |
| Occupation 1 | 0.42 | 0.06 | 0.09 | NA |
| Occupation 2 | **0.58** | **0.66** | **0.88** | NA |
| Occupation 3 | 0.01 | 0.28 | 0.02 | NA |
| Education 1 | **0.6** | 0 | 0.18 | NA |
| Education 2 | 0.17 | 0.04 | 0.35 | NA |
| Education 3 | 0.23 | **0.96** | **0.47** | NA |
| Insurance 1 | 0.24 | 0.02 | 0.07 | NA |
| Insurance 2 | **0.73** | 0.14 | 0.32 | NA |
| Insurance 3 | 0.04 | **0.84** | **0.61** | NA |
| Four-latent-class solution | | | | |
| Mean PP | 0.12 | 0.29 | 0.22 | 0.37 |
| Prevalence | 0.23 | 0.31 | 0.17 | 0.29 |
| Income 1 | 0.28 | 0.03 | **0.75** | 0.17 |
| Income 2 | **0.49** | 0.19 | 0.25 | **0.63** |
| Income 3 | 0.23 | **0.78** | 0 | 0.2 |
| Occupation 1 | 0.22 | 0.06 | 0.42 | 0.01 |
| Occupation 2 | **0.72** | **0.68** | **0.58** | **0.99** |
| Occupation 3 | 0.06 | 0.26 | 0 | 0 |
| Education 1 | 0.28 | 0 | **0.6** | 0.14 |
| Education 2 | 0.26 | 0.05 | 0.18 | 0.42 |
| Education 3 | **0.47** | **0.95** | 0.22 | **0.45** |
| Insurance 1 | 0.2 | 0.01 | 0.2 | 0.01 |
| Insurance 2 | 0.23 | 0.15 | **0.8** | 0.37 |
| Insurance 3 | **0.57** | **0.84** | 0 | **0.62** |

Mean PP = mean posterior probability; NA = not available

* The maximal item-response probabilities for each latent class were marked in bold.

† Prevalence indicated the prevalence of each latent class. Income 1 to 3 respectively referred to the poverty income ratio of ≥3.5, ≥1.3 to <3.5, and <1.3. Occupation 1 to 3 respectively referred to upper socioeconomic index, lower socioeconomic index, and unemployment. Education 1 to 3 respectively referred to college or above, high school or equivalent, less than high school. Insurance 1 to 3 respectively referred to private health insurance, public health insurance only, and no health insurance.

**Supplementary Table 3** The mediating proportion of mean AL on the association between SES and CERAD.

|  | Direct Effect | | Indirect Effect | | Total Effect | | Proportion Mediated (%) |
| --- | --- | --- | --- | --- | --- | --- | --- |
|  | β (95%CI) | P-Value | β (95%CI) | P-Value | β (95%CI) | P-Value |  |
| Sex |  |  |  |  |  |  |  |
| Male | -1.76 (-2.61 to -1.06) | < 0.01 | -0.02 (-0.21 to 0.2) | 0.44 | -1.78 (-2.68 to -1.1) | < 0.01 | - |
| Female | -1.39 (-2.29 to -0.65) | < 0.01 | -0.1 (-0.28 to 0.06) | 0.09 | -1.49 (-2.4 to -0.85) | < 0.01 | - |
| Age |  |  |  |  |  |  |  |
| 60 - 69 | -1.78 (-2.65 to -1.01) | < 0.01 | 0.05 (-0.12 to 0.3) | 0.72 | -1.72 (-2.62 to -1.04) | < 0.01 | - |
| 70 - 79 | -1.35 (-2.67 to -0.34) | < 0.01 | -0.11 (-0.35 to 0.03) | 0.07 | -1.44 (-2.7 to -0.6) | < 0.01 | - |
| ≥80 | -0.22 (-2.03 to 1.96) | 0.39 | -0.15 (-0.54 to 0.13) | 0.14 | -0.4 (-2.2 to 1.57) | 0.28 | - |
| BMI |  |  |  |  |  |  |  |
| < 25 | -1.8 (-2.96 to -0.77) | < 0.01 | -0.23 (-0.62 to 0.02) | 0.03 | -1.99 (-3.29 to -1.04) | < 0.01 | 11.49% |
| 25 to <30 | -1.43 (-2.51 to -0.57) | < 0.01 | -0.01 (-0.22 to 0.23) | 0.48 | -1.44 (-2.51 to -0.58) | < 0.01 | - |
| ≥30 | -1.78 (-3.21 to -0.99) | < 0.01 | 0.04 (-0.15 to 0.3) | 0.64 | -1.73 (-3 to -0.98) | < 0.01 | - |
| Race |  |  |  |  |  |  |  |
| NHB | -1.01 (-2.1 to -0.12) | 0.01 | 0.11 (-0.02 to 0.3) | 0.96 | -0.9 (-1.86 to -0.13) | 0.01 | - |
| NHW | -1.53 (-2.41 to -0.78) | < 0.01 | -0.08 (-0.29 to 0.13) | 0.21 | -1.61 (-2.49 to -0.82) | < 0.01 | - |
| Other | -1.5 (-2.33 to -0.83) | < 0.01 | -0.12 (-0.31 to 0.03) | 0.05 | -1.63 (-2.45 to -1) | < 0.01 | - |
| Smoking, n (%) | |  |  |  |  |  |  |
| No | -1.32 (-2.11 to -0.69) | < 0.01 | -0.03 (-0.2 to 0.16) | 0.35 | -1.35 (-2.11 to -0.72) | < 0.01 | - |
| Yes | -1.63 (-2.6 to -0.82) | < 0.01 | -0.05 (-0.25 to 0.13) | 0.29 | -1.68 (-2.62 to -0.9) | < 0.01 | - |
| Alcoholic drinks, n (%) | |  |  |  |  |  |  |
| No | -1.49 (-2.75 to -0.54) | < 0.01 | -0.02 (-0.17 to 0.11) | 0.35 | -1.5 (-2.69 to -0.62) | < 0.01 | - |
| Yes | -1.54 (-2.29 to -0.97) | < 0.01 | -0.09 (-0.27 to 0.09) | 0.15 | -1.63 (-2.34 to -1.08) | < 0.01 | - |
| Physical activity, n (%) | |  |  |  |  |  |  |
| Vigorous or moderate | -1.61 (-2.33 to -0.93) | < 0.01 | -0.1 (-0.28 to 0.1) | 0.14 | -1.7 (-2.38 to -1.15) | < 0.01 | - |
| Other | -1.66 (-2.82 to -0.82) | < 0.01 | -0.05 (-0.25 to 0.12) | 0.25 | -1.71 (-2.91 to -0.87) | < 0.01 | - |

**Supplementary Table 4** The mediating proportion of mean AL on the association between SES and AFT.

|  | Direct Effect | | Indirect Effect | | Total Effect | | Proportion Mediated (%) |
| --- | --- | --- | --- | --- | --- | --- | --- |
|  | β (95%CI) | P-Value | β (95%CI) | P-Value | β (95%CI) | P-Value |  |
| Sex |  |  |  |  |  |  |  |
| Male | -1.26 (-2.13 to -0.67) | < 0.01 | -0.14 (-0.3 to 0.02) | 0.04 | -1.39 (-2.22 to -0.74) | < 0.01 | 9.80% |
| Female | -1.4 (-2.27 to -0.63) | < 0.01 | -0.02 (-0.27 to 0.27) | 0.47 | -1.42 (-2.33 to -0.71) | < 0.01 | - |
| Age |  |  |  |  |  |  |  |
| 60 - 69 | -1.4 (-2.17 to -0.81) | < 0.01 | -0.11 (-0.35 to 0.13) | 0.2 | -1.51 (-2.21 to -0.9) | < 0.01 | - |
| 70 - 79 | -1.11 (-2.45 to -0.23) | 0.02 | -0.06 (-0.24 to 0.1) | 0.21 | -1.16 (-2.54 to -0.32) | 0.01 | - |
| ≥80 | -1.58 (-4.21 to -0.05) | 0.02 | 0.04 (-0.39 to 0.58) | 0.53 | -1.54 (-4.56 to -0.03) | 0.02 | - |
| BMI |  |  |  |  |  |  |  |
| < 25 | -0.97 (-2.58 to -0.25) | 0.01 | 0.03 (-0.36 to 0.34) | 0.42 | -0.94 (-2.57 to -0.27) | < 0.01 | - |
| 25 to <30 | -0.97 (-1.97 to -0.23) | 0.01 | 0.03 (-0.21 to 0.32) | 0.62 | -0.94 (-1.86 to -0.18) | 0.01 | - |
| ≥30 | -2.38 (-3.79 to -1.48) | < 0.01 | -0.12 (-0.38 to 0.09) | 0.14 | -2.49 (-3.91 to -1.55) | < 0.01 | - |
| Race |  |  |  |  |  |  |  |
| NHB | -1.07 (-1.98 to -0.35) | < 0.01 | 0.08 (-0.04 to 0.25) | 0.91 | -0.98 (-1.89 to -0.29) | < 0.01 | - |
| NHW | -1.21 (-2.22 to -0.27) | 0.01 | -0.16 (-0.44 to 0.11) | 0.1 | -1.39 (-2.37 to -0.56) | < 0.01 | - |
| Other | -1.39 (-2.13 to -0.83) | < 0.01 | -0.08 (-0.25 to 0.09) | 0.17 | -1.46 (-2.22 to -0.9) | < 0.01 | - |
| Smoking, n (%) | |  |  |  |  |  |  |
| No | -1.72 (-2.58 to -1.11) | < 0.01 | 0 (-0.18 to 0.16) | 0.47 | -1.72 (-2.51 to -1.1) | < 0.01 | - |
| Yes | -0.86 (-1.84 to -0.04) | 0.02 | -0.14 (-0.36 to 0.08) | 0.13 | -1.01 (-1.91 to -0.28) | < 0.01 | - |
| Alcoholic drinks, n (%) | |  |  |  |  |  |  |
| No | -1.83 (-2.78 to -1.01) | < 0.01 | -0.07 (-0.26 to 0.07) | 0.15 | -1.9 (-2.77 to -1.14) | < 0.01 | - |
| Yes | -1.25 (-2.01 to -0.62) | < 0.01 | -0.08 (-0.29 to 0.15) | 0.26 | -1.34 (-2.05 to -0.73) | < 0.01 | - |
| Physical activity, n (%) | |  |  |  |  |  |  |
| Vigorous or moderate | -1.41 (-2.12 to -0.79) | < 0.01 | -0.05 (-0.26 to 0.2) | 0.36 | -1.46 (-2.15 to -0.87) | < 0.01 | - |
| Other | -1.32 (-2.5 to -0.64) | < 0.01 | -0.14 (-0.35 to 0.07) | 0.07 | -1.45 (-2.61 to -0.63) | < 0.01 | - |

**Supplementary Table 5** The mediating proportion of mean AL on the association between SES and DSST.

|  | Direct Effect | | Indirect Effect | | Total Effect | | Proportion Mediated (%) |
| --- | --- | --- | --- | --- | --- | --- | --- |
|  | β (95%CI) | P-Value | β (95%CI) | P-Value | β (95%CI) | P-Value |  |
| Sex |  |  |  |  |  |  |  |
| Male | -2.63 (-3.83 to -1.82) | < 0.01 | -0.31 (-0.59 to -0.1) | < 0.01 | -2.88 (-4.06 to -2.07) | < 0.01 | 10.77% |
| Female | -1.92 (-3.16 to -1.23) | < 0.01 | -0.31 (-0.55 to -0.12) | < 0.01 | -2.18 (-3.3 to -1.44) | < 0.01 | 14.09% |
| Age |  |  |  |  |  |  |  |
| 60 - 69 | -3.32 (-5.06 to -2.5) | < 0.01 | -0.23 (-0.47 to -0.03) | 0.02 | -3.55 (-5.39 to -2.68) | < 0.01 | 6.37% |
| 70 - 79 | -2.12 (-4.16 to -1.28) | < 0.01 | -0.3 (-0.68 to -0.07) | < 0.01 | -2.28 (-4.2 to -1.48) | < 0.01 | 12.93% |
| ≥80 | -1.07 (-3.3 to 0.57) | 0.1 | -0.23 (-0.7 to 0.09) | 0.08 | -1.34 (-3.4 to 0.42) | 0.05 | - |
| BMI |  |  |  |  |  |  |  |
| < 25 | -1.89 (-3.5 to -0.89) | < 0.01 | -0.3 (-0.63 to 0.05) | 0.05 | -2.17 (-3.76 to -1.16) | < 0.01 | 13.93% |
| 25 to <30 | -2.53 (-4.76 to -1.64) | < 0.01 | -0.33 (-0.67 to -0.11) | < 0.01 | -2.65 (-4.57 to -1.79) | < 0.01 | 12.62% |
| ≥30 | -2.8 (-4.74 to -1.94) | < 0.01 | -0.33 (-0.69 to -0.12) | < 0.01 | -3.11 (-5.25 to -2.27) | < 0.01 | 10.59% |
| Race |  |  |  |  |  |  |  |
| NHB | -2.15 (-3.44 to -1.42) | < 0.01 | -0.1 (-0.31 to 0.05) | 0.08 | -2.26 (-3.59 to -1.59) | < 0.01 | - |
| NHW | -1.73 (-3.22 to -0.76) | < 0.01 | -0.36 (-0.69 to -0.11) | < 0.01 | -2.1 (-3.28 to -1.13) | < 0.01 | 17.20% |
| Other | -3.83 (-5.93 to -3.03) | < 0.01 | -0.36 (-0.63 to -0.16) | < 0.01 | -4.06 (-6.14 to -3.27) | < 0.01 | 8.89% |
| Smoking, n (%) | |  |  |  |  |  |  |
| No | -2.38 (-3.57 to -1.67) | < 0.01 | -0.31 (-0.58 to -0.09) | < 0.01 | -2.61 (-3.7 to -1.97) | < 0.01 | 11.69% |
| Yes | -2.26 (-3.8 to -1.38) | < 0.01 | -0.31 (-0.55 to -0.11) | < 0.01 | -2.57 (-4.04 to -1.73) | < 0.01 | 12.20% |
| Alcoholic drinks, n (%) | |  |  |  |  |  |  |
| No | -1.71 (-3.18 to -0.87) | < 0.01 | -0.17 (-0.45 to -0.03) | 0.01 | -1.88 (-3.43 to -1.07) | < 0.01 | 8.82% |
| Yes | -2.72 (-3.8 to -1.96) | < 0.01 | -0.38 (-0.61 to -0.19) | < 0.01 | -3.04 (-4.15 to -2.29) | < 0.01 | 12.45% |
| Physical activity, n (%) | |  |  |  |  |  |  |
| Vigorous or moderate | -2.38 (-3.45 to -1.69) | < 0.01 | -0.26 (-0.5 to -0.09) | < 0.01 | -2.62 (-3.72 to -1.94) | < 0.01 | 10.06% |
| Other | -2.29 (-3.73 to -1.51) | < 0.01 | -0.38 (-0.67 to -0.17) | < 0.01 | -2.53 (-3.92 to -1.7) | < 0.01 | 15.04% |

**Supplementary Table 6** The mediating proportion of mean PD on the association between SES and CERAD.

|  | Direct Effect | | Indirect Effect | | Total Effect | | Proportion Mediated (%) |
| --- | --- | --- | --- | --- | --- | --- | --- |
|  | β (95%CI) | P-Value | β (95%CI) | P-Value | β (95%CI) | P-Value |  |
| Sex |  |  |  |  |  |  |  |
| Male | -1.76 (-2.68 to -1.09) | < 0.01 | -0.01 (-0.14 to 0.13) | 0.42 | -1.78 (-2.66 to -1.1) | < 0.01 | - |
| Female | -1.39 (-2.37 to -0.7) | < 0.01 | -0.1 (-0.27 to 0.02) | 0.06 | -1.49 (-2.45 to -0.8) | < 0.01 | - |
| Age |  |  |  |  |  |  |  |
| 60 - 69 | -1.74 (-2.61 to -1.09) | < 0.01 | 0.02 (-0.13 to 0.23) | 0.61 | -1.72 (-2.66 to -1.07) | < 0.01 | - |
| 70 - 79 | -1.41 (-2.71 to -0.51) | < 0.01 | -0.04 (-0.21 to 0.1) | 0.28 | -1.44 (-2.77 to -0.55) | < 0.01 | - |
| ≥80 | -0.36 (-2.16 to 1.71) | 0.3 | -0.03 (-0.27 to 0.13) | 0.36 | -0.4 (-2.23 to 1.6) | 0.29 | - |
| BMI |  |  |  |  |  |  |  |
| < 25 | -1.82 (-3.1 to -0.74) | < 0.01 | -0.2 (-0.52 to 0) | 0.03 | -1.99 (-3.24 to -1.03) | < 0.01 | 10.28% |
| 25 to <30 | -1.47 (-2.59 to -0.67) | < 0.01 | 0.02 (-0.07 to 0.18) | 0.71 | -1.44 (-2.56 to -0.6) | < 0.01 | - |
| ≥30 | -1.69 (-2.94 to -0.85) | < 0.01 | -0.04 (-0.22 to 0.12) | 0.28 | -1.73 (-2.92 to -0.9) | < 0.01 | - |
| Race |  |  |  |  |  |  |  |
| NHB | -1 (-1.96 to -0.18) | 0.01 | 0.07 (-0.03 to 0.26) | 0.93 | -0.9 (-1.87 to -0.12) | 0.01 | - |
| NHW | -1.51 (-2.41 to -0.72) | < 0.01 | -0.1 (-0.26 to 0.03) | 0.06 | -1.61 (-2.51 to -0.92) | < 0.01 | - |
| Other | -1.53 (-2.3 to -0.82) | < 0.01 | -0.09 (-0.28 to 0.09) | 0.16 | -1.63 (-2.43 to -0.9) | < 0.01 | - |
| Smoking, n (%) | |  |  |  |  |  |  |
| No | -1.31 (-2.14 to -0.66) | < 0.01 | -0.05 (-0.2 to 0.08) | 0.22 | -1.35 (-2.18 to -0.75) | < 0.01 | - |
| Yes | -1.64 (-2.71 to -0.86) | < 0.01 | -0.04 (-0.18 to 0.1) | 0.25 | -1.68 (-2.71 to -0.92) | < 0.01 | - |
| Alcoholic drinks, n (%) | |  |  |  |  |  |  |
| No | -1.48 (-2.57 to -0.58) | < 0.01 | -0.03 (-0.15 to 0.08) | 0.28 | -1.5 (-2.61 to -0.65) | < 0.01 | - |
| Yes | -1.56 (-2.25 to -0.94) | < 0.01 | -0.07 (-0.22 to 0.05) | 0.13 | -1.63 (-2.42 to -1.08) | < 0.01 | - |
| Physical activity, n (%) | |  |  |  |  |  |  |
| Vigorous or moderate | -1.62 (-2.34 to -1.04) | < 0.01 | -0.1 (-0.24 to 0) | 0.03 | -1.7 (-2.41 to -1.11) | < 0.01 | 5.81% |
| Other | -1.73 (-2.95 to -0.88) | < 0.01 | 0.02 (-0.14 to 0.21) | 0.6 | -1.71 (-2.86 to -0.89) | < 0.01 | - |

**Supplementary Table 7** The mediating proportion of mean PD on the association between SES and AFT.

|  | Direct Effect | | Indirect Effect | | Total Effect | | Proportion Mediated (%) |
| --- | --- | --- | --- | --- | --- | --- | --- |
|  | β (95%CI) | P-Value | β (95%CI) | P-Value | β (95%CI) | P-Value |  |
| Sex |  |  |  |  |  |  |  |
| Male | -1.44 (-2.33 to -0.74) | < 0.01 | 0.02 (-0.11 to 0.21) | 0.64 | -1.42 (-2.25 to -0.72) | < 0.01 | - |
| Female | -1.39 (-2.2 to -0.78) | < 0.01 | 0 (-0.16 to 0.17) | 0.54 | -1.39 (-2.27 to -0.77) | < 0.01 | - |
| Age |  |  |  |  |  |  |  |
| 60 - 69 | -1.55 (-2.26 to -0.95) | < 0.01 | 0.05 (-0.12 to 0.23) | 0.7 | -1.51 (-2.28 to -0.9) | < 0.01 | - |
| 70 - 79 | -1.15 (-2.49 to -0.21) | 0.01 | -0.01 (-0.12 to 0.07) | 0.39 | -1.16 (-2.54 to -0.27) | 0.01 | - |
| ≥80 | -1.56 (-4.18 to -0.13) | 0.01 | 0.02 (-0.2 to 0.19) | 0.56 | -1.54 (-4.05 to -0.15) | 0.01 | - |
| BMI |  |  |  |  |  |  |  |
| < 25 | -1.49 (-2.81 to -0.58) | < 0.01 | 0.02 (-0.23 to 0.3) | 0.53 | -1.47 (-2.84 to -0.62) | < 0.01 | - |
| 25 to <30 | -1 (-1.98 to -0.23) | 0.01 | 0.05 (-0.05 to 0.21) | 0.85 | -0.94 (-1.91 to -0.11) | 0.01 | - |
| ≥30 | -2.43 (-3.74 to -1.54) | < 0.01 | -0.06 (-0.27 to 0.12) | 0.23 | -2.49 (-3.95 to -1.6) | < 0.01 | - |
| Race |  |  |  |  |  |  |  |
| NHB | -1.04 (-1.91 to -0.38) | < 0.01 | 0.04 (-0.02 to 0.14) | 0.87 | -0.98 (-1.87 to -0.28) | < 0.01 | - |
| NHW | -1.39 (-2.38 to -0.46) | < 0.01 | 0.01 (-0.18 to 0.22) | 0.5 | -1.39 (-2.4 to -0.51) | < 0.01 | - |
| Other | -1.4 (-2.11 to -0.81) | < 0.01 | -0.07 (-0.27 to 0.1) | 0.21 | -1.46 (-2.2 to -0.92) | < 0.01 | - |
| Smoking, n (%) | |  |  |  |  |  |  |
| No | -1.78 (-2.6 to -1.2) | < 0.01 | 0.05 (-0.08 to 0.19) | 0.81 | -1.72 (-2.53 to -1.07) | < 0.01 | - |
| Yes | -1 (-1.93 to -0.31) | < 0.01 | -0.01 (-0.17 to 0.16) | 0.44 | -1.01 (-1.94 to -0.3) | 0.01 | - |
| Alcoholic drinks, n (%) | |  |  |  |  |  |  |
| No | -1.9 (-2.88 to -1.06) | < 0.01 | 0 (-0.14 to 0.12) | 0.47 | -1.9 (-2.83 to -1.13) | < 0.01 | - |
| Yes | -1.37 (-2.1 to -0.77) | < 0.01 | 0.03 (-0.11 to 0.18) | 0.67 | -1.34 (-2.07 to -0.75) | < 0.01 | - |
| Physical activity, n (%) | |  |  |  |  |  |  |
| Vigorous or moderate | -1.51 (-2.15 to -0.93) | < 0.01 | 0.04 (-0.1 to 0.22) | 0.73 | -1.46 (-2.19 to -0.87) | < 0.01 | - |
| Other | -1.43 (-2.64 to -0.68) | < 0.01 | -0.02 (-0.19 to 0.16) | 0.4 | -1.45 (-2.61 to -0.73) | < 0.01 | - |

**Supplementary Table 8** The mediating proportion of mean PD on the association between SES and DSST.

|  | Direct Effect | | Indirect Effect | | Total Effect | | Proportion Mediated (%) |
| --- | --- | --- | --- | --- | --- | --- | --- |
|  | β (95%CI) | P-Value | β (95%CI) | P-Value | β (95%CI) | P-Value |  |
| Sex |  |  |  |  |  |  |  |
| Male | -2.75 (-4.09 to -2.01) | < 0.01 | -0.15 (-0.33 to -0.01) | 0.02 | -2.88 (-4.1 to -2.15) | < 0.01 | 5.34% |
| Female | -2.06 (-3.23 to -1.36) | < 0.01 | -0.19 (-0.38 to -0.05) | < 0.01 | -2.18 (-3.47 to -1.44) | < 0.01 | 8.77% |
| Age |  |  |  |  |  |  |  |
| 60 - 69 | -3.39 (-5.33 to -2.63) | < 0.01 | -0.16 (-0.33 to 0.01) | 0.03 | -3.55 (-5.37 to -2.66) | < 0.01 | 4.41% |
| 70 - 79 | -2.33 (-4.1 to -1.38) | < 0.01 | -0.05 (-0.3 to 0.12) | 0.27 | -2.28 (-4.15 to -1.46) | < 0.01 | - |
| ≥80 | -1.3 (-3.41 to 0.09) | 0.03 | -0.03 (-0.29 to 0.13) | 0.36 | -1.34 (-3.36 to 0.17) | 0.04 | - |
| BMI |  |  |  |  |  |  |  |
| < 25 | -2.11 (-3.83 to -1.06) | < 0.01 | -0.05 (-0.31 to 0.23) | 0.35 | -2.17 (-3.6 to -1.15) | < 0.01 | - |
| 25 to <30 | -2.64 (-4.75 to -1.83) | < 0.01 | -0.07 (-0.25 to 0.03) | 0.11 | -2.65 (-4.56 to -1.82) | < 0.01 | - |
| ≥30 | -2.96 (-5.26 to -2.22) | < 0.01 | -0.31 (-0.57 to -0.11) | < 0.01 | -3.11 (-5.04 to -2.27) | < 0.01 | 9.84% |
| Race |  |  |  |  |  |  |  |
| NHB | -2.24 (-3.56 to -1.6) | < 0.01 | -0.01 (-0.11 to 0.06) | 0.33 | -2.26 (-3.55 to -1.56) | < 0.01 | - |
| NHW | -1.9 (-3.13 to -0.9) | < 0.01 | -0.25 (-0.51 to -0.04) | 0.01 | -2.1 (-3.43 to -1.19) | < 0.01 | 11.66% |
| Other | -3.87 (-5.86 to -3.1) | < 0.01 | -0.24 (-0.47 to -0.06) | < 0.01 | -4.06 (-6.17 to -3.14) | < 0.01 | 5.83% |
| Smoking, n (%) | |  |  |  |  |  |  |
| No | -2.5 (-3.62 to -1.83) | < 0.01 | -0.17 (-0.37 to -0.03) | 0.01 | -2.61 (-3.74 to -1.94) | < 0.01 | 6.32% |
| Yes | -2.42 (-4.11 to -1.57) | < 0.01 | -0.19 (-0.37 to -0.05) | 0.01 | -2.57 (-4.06 to -1.78) | < 0.01 | 7.21% |
| Alcoholic drinks, n (%) | |  |  |  |  |  |  |
| No | -1.8 (-3.17 to -0.92) | < 0.01 | -0.1 (-0.3 to 0.02) | 0.06 | -1.88 (-3.38 to -1.01) | < 0.01 | - |
| Yes | -2.89 (-3.95 to -2.16) | < 0.01 | -0.19 (-0.34 to -0.06) | < 0.01 | -3.04 (-4.14 to -2.3) | < 0.01 | 6.38% |
| Physical activity, n (%) | |  |  |  |  |  |  |
| Vigorous or moderate | -2.51 (-3.6 to -1.88) | < 0.01 | -0.15 (-0.32 to -0.02) | 0.01 | -2.62 (-3.66 to -1.94) | < 0.01 | 5.79% |
| Other | -2.37 (-3.85 to -1.55) | < 0.01 | -0.24 (-0.46 to -0.09) | < 0.01 | -2.53 (-3.94 to -1.7) | < 0.01 | 9.62% |
